# Modality-specific impacts of distractors on visual and auditory categorical decision-making: an evidence accumulation perspective

**DOI:** 10.3389/fpsyg.2024.1380196

**Published:** 2024-05-03

**Authors:** Jianhua Li, Lin Hua, Sophia W. Deng

**Affiliations:** ^1^Department of Psychology, University of Macau, Macau, China; ^2^Center for Cognitive and Brain Sciences, University of Macau, Macau, China; ^3^Faculty of Health Sciences, University of Macau, Macau, China

**Keywords:** multisensory perception, categorical decision-making, evidence accumulation processes, EEG decoding, hierarchical drift diffusion modeling

## Abstract

Our brain constantly processes multisensory inputs to make decisions and guide behaviors, but how goal-relevant processes are influenced by irrelevant information is unclear. Here, we investigated the effects of intermodal and intramodal task-irrelevant information on visual and auditory categorical decision-making. In both visual and auditory tasks, we manipulated the modality of irrelevant inputs (visual vs. auditory vs. none) and used linear discrimination analysis of EEG and hierarchical drift-diffusion modeling (HDDM) to identify when and how task-irrelevant information affected decision-relevant processing. The results revealed modality-specific impacts of irrelevant inputs on visual and auditory categorical decision-making. The distinct effects on the visual task were shown on the neural components, with auditory distractors amplifying the sensory processing whereas visual distractors amplifying the post-sensory process. Conversely, the distinct effects on the auditory task were shown in behavioral performance and underlying cognitive processes. Visual distractors facilitate behavioral performance and affect both stages, but auditory distractors interfere with behavioral performance and impact on the sensory processing rather than the post-sensory decision stage. Overall, these findings suggested that auditory distractors affect the sensory processing stage of both tasks while visual distractors affect the post-sensory decision stage of visual categorical decision-making and both stages of auditory categorical decision-making. This study provides insights into how humans process information from multiple sensory modalities during decision-making by leveraging modality-specific impacts.

## 1 Introduction

We are constantly bombarded with information from multiple senses and make categorical decisions based on the features of the inputs to simplify our understanding of the world. In such complex multisensory scenarios, some of these inputs are task-relevant while some are less relevant. Research showed that our categorization performance can be facilitated or hindered by task-irrelevant information and the modality of inputs could modulate the impacts (see the review, Li and Deng, 2023a). In the cognitive process of categorical decision-making, our brain accumulates information from various sensors over time and ultimately elicits a motor response when a decision criterion is reached (Gold and Shadlen, 2007; Bizley et al., 2016; Keil and Senkowski, 2018; Trueblood et al., 2021). However, from the framework of evidence accumulation, it remains unclear about the precise effects of irrelevant information on the evidence accumulation of goal-relevant processes. Furthermore, the impact of input modality on categorical decision-making remains unclear, as previous research has primarily focused on how categorical evidence accumulated in the visual modality (e.g., Diaz et al., 2017; Kayser et al., 2017; Franzen et al., 2020). Here, through identifying neural activities and modeling the latent cognitive processes, we examined how intramodal and intermodal distractors (task-irrelevant or distracting information, Wöstmann et al., 2022) affect visual and auditory categorization that tap into different stages of evidence accumulation.

### 1.1 Impacts of distractors on evidence accumulation process: sensory processing account vs. post-sensory decision account

Impacts of task-irrelevant information on evidence accumulation of decision-making can be linked at either the sensory processing level (Schroeder and Foxe, 2005; Kayser and Logothetis, 2007) or the post-sensory decision level (Franzen et al., 2020; Merz et al., 2021). The early processing hypothesis posits that multiple pieces of evidence are combined at the sensory processing stage (Kayser and Logothetis, 2007; Jensen et al., 2019), which is supported by findings showing that (1) early attentional allocation or the early saliency computation was influenced (Geyer et al., 2010); (2) different activations in the primary sensory cortex for perceptual representation, rather than the higher areas for decision execution was elicited (Johnson et al., 2007; Geyer et al., 2010); and (3) temporally distinct EEG component occurred early after stimulus presentation (Philiastides and Sajda, 2006b; Philiastides et al., 2006). In contrast, the late processing hypothesis assumes that evidence is accumulated independently at the early sensory encoding, and interplays at the post-sensory decision phase (Bizley et al., 2016; Aller and Noppeney, 2019). For example, visual and auditory evidence was accumulated in the visual primary and auditory primary cortex independently and then combined for a decision response at the decision-making stage in higher cortices (Aller and Noppeney, 2019).

To characterize the evidence accumulation processes underlying behavioral performance, sequential-sampling models including the drift-diffusion model (DDM) have been applied to address at which cognitive processes, task-irrelevant information operates (Franzen et al., 2020; Chen et al., 2021). Such models assume that over the course of decision-making, people continuously sample and gradually accumulate information until a threshold of evidence is reached (Ratcliff and McKoon, 2008). The sensory processing account is modeled as associated with a bias in the rate of evidence accumulation, which should be inferred from the phenomenon that compared with the no-distractor trials, information accumulates faster or slower when with irrelevant inputs. Conversely, the post-sensory decision account could be correlated with a bias in the response boundary, as this account assumed that the higher-level information integration process was affected. Task-irrelevant information in this account changes the response threshold, reducing or increasing the amount of evidence required to make a response. Nevertheless, this model is a more sensitive way than behavioral indicators to look into what changed by a task-irrelevant input in evidence accumulation processes for decision-making (Ratcliff et al., 2016).

In addition, the impacts of distractors on the sensory processing stage and post-sensory decision stage can be dissociated by time-resolved electroencephalography (EEG) (Talsma and Woldorff, 2005; Philiastides et al., 2006; Kayser et al., 2017; Franzen et al., 2020), which are marked by early and late neural components, respectively. Some studies showed that the late component, rather than the early component, better predicted the influence of the task-irrelevant or less-relevant information on the goal-relevant information processing (Li et al., 2010; Kayser et al., 2017; Franzen et al., 2020), whereas some findings suggest that multiple signals accelerate both stages (Mercier and Cappe, 2020). However, less is known about the role of sensory modalities in the influence of distractors on the evidence accumulation of goal-relevant processing. Distinguishing between the impacts of distractors on the sensory processing stage and post-sensory decision stage is critical for a detailed understanding of how categorical decisions are formed based on perceptual-level multisensory context and higher-level conceptualization of evidence. This distinction is crucial as it reveals a general neural mechanism of distractor processing in decision-making across modalities, thereby facilitating the deciphering of the mechanisms underlying multisensory integration.

### 1.2 The modulation of input modality

Previous research has investigated visual perceptual decision-making, focusing on some types of basic features like color (Jensen et al., 2019), shape (Giard and Peronnet, 1999), facial expression (Young, 2016), and random-dot motion (Kayser et al., 2017). However, there is limited understanding regarding more complex decision-making and decision-making based on auditory inputs. Auditory decision-making encompasses various tasks, such as the recognition of spoken words or phrases in different languages (Samuel and Kraljic, 2009), the differentiation of different emotional states conveyed through vocal cues (Bachorowski, 1999), and the differentiation of musical instruments based on their sound characteristics (Reetzke et al., 2016). In the real world, decisions are often complex, needing evidence accumulation from multiple modalities or from stimuli separated in time and space, and requiring judgments on which may or may not be task-relevant. The interest in studying more complex decision-making tasks, such as categorical decision-making under multisensory contexts has increased. For example, Li and Deng (2023a) conducted a meta-analysis on multisensory learning research in the last two decades and revealed the facilitation and interference effects of cross-modal distractors on target processing. The results also suggested the moderating effects of modality on multisensory learning and pointed out that the underlying mechanism remained unknown. Since auditory and visual inputs are processed differently (Stein, 2012; Wilson et al., 2015), with auditory stimuli usually being dynamic, transient, and presented sequentially whereas visual stimuli typically presented simultaneously, we predict that a modality-dependent effect of irrelevant inputs on relevant information processing could be observed.

It has been demonstrated that visual modality is dominant in the spatial domain, while auditory modality is relatively specialized in temporal processing (Freides, 1974; Repp and Penel, 2002; Koelewijn et al., 2010). Therefore, to effectively investigate the influence of irrelevant information processing on task-relevant information processing, it is advisable to present the visual and auditory targets in their optimal modes: simultaneous presentation for the visual target and sequential presentation for the auditory target. For example, Mcauley et al. (2006) found that participants were better able to detect the regularity or predictability of events over time in an auditory sequence (e.g., a series of beeps) compared to a visual sequence (e.g., a series of flashing lights), even when the two sequences were matched for complexity. Similarly, Repp and Penel (2002) found that participants were better at synchronizing finger tapping with an auditory metronome than with a visual metronome, indicating that temporal information was accumulated more accurately by the auditory modality than by the visual modality.

In the current study, we aimed to understand how intramodal and intermodal distractors affect visual categorical decision-making, as well as their influence on auditory categorical decision-making, from the perspective of evidence accumulation. Considering the spatial separation of visual inputs and temporal separation of auditory inputs, task-relevant information with multiple items was presented simultaneously in the visual categorization task and sequentially in auditory categorization. The main research questions of this study were threefold. First, how does visual and auditory irrelevant information affect the evidence accumulation of visual categorical decision-making in which task-relevant items were presented simultaneously? Second, how does visual and auditory irrelevant information affect the evidence accumulation of auditory categorical decision-making in which task-relevant items were presented sequentially? Finally, do visual and auditory distractors have similar or different effects on evidence accumulation for visual or auditory categorical decision-making?

### 1.3 The present study

Given the research gap on auditory decision-making and the two stages in decision-making (sensory processing stage and post-sensory decision stage) reviewed above, the effects of intermodal and intramodal distractors on visual and auditory categorization could occur in sensory processing stage only, in post-sensory decision stage only, or in both stages. Considering the distinct processing of auditory and visual inputs (Stein, 2012; Wilson et al., 2015) and the modulation of inputs’ modalities on categorical decision-making (Li and Deng, 2023a), we predicted a modality-specific impact. Specifically, the impact of distractors on the stages of categorical decision-making varied based on the modality of the inputs. For the visual categorical decision-making, previous research showed that auditory distractors could lead to a lower evidence accumulation rate without affecting the response boundary (Philiastides and Ratcliff, 2013). Additionally, auditory distractors are alert and transient (Stein, 2012) and would be prioritized over task-relevant information processing. Based on these findings, we predicted that auditory distractors would primarily impact the sensory processing rather than the post-sensory decision stage. In contrast, visual information is often processed in a parallel and spatially distributed manner (Nassi and Callaway, 2009). The task-irrelevant information would be accumulated after the task-relevant information. Consequently, it was hypothesized that visual distractors would mainly affect the post-sensory decision stage. For the auditory categorical decision-making, there is limited research on how irrelevant information affects decision-making from an evidence accumulation perspective. It is difficult to accurately predict the stage at which irrelevant information will influence decision-making. Nevertheless, like the effect on visual categorical decision-making, there could be a modality-specific impact.

To test our hypothesis, we employed two experiments (within participants), a visual categorical decision-making task and an auditory categorical decision-making task, in which categorical features of task-relevant stimuli were provided simultaneously (visual) and sequentially (auditory). Crucially, to examine the impacts of visual and auditory task-irrelevant information, participants were required to finish three conditions of each task (no-distractor, visual distractor, and auditory distractor). In the distractor conditions of both tasks, there was one distractor on each trial, and the visual or auditory distractor was presented until participants made a response. We used HDDM and EEG technique to explore and compare the impacts of visual and auditory irrelevant inputs on behavior performance, modeling parameters, and neural signals during visual or auditory categorical decision-making.

## 2 Materials and methods

### 2.1 Participants

Forty healthy volunteers, right-handed with self-reported normal hearing and normal or corrected-to-normal vision, were recruited from the University of Macau. They were tested on both visual and auditory categorical decision-making tasks, and one of them was excluded due to not completing both tasks. The final sample consisted of 39 volunteers (11 males, *M* = 21.54 years, *SD* = 1.85 years, range 19–25 years). All of them were naive about the hypotheses of the study and were tested in a sound-attenuated and electrostatically shielded room in the psychology laboratory at the University of Macau. Before starting the task, a written consent form was provided.

### 2.2 Stimuli

In the visual task, the task-relevant stimuli were colorful images of seven animals (bird, dog, pig, sheep, chick, cow, and cat), with 200 × 200 pixels. In the auditory task, the task-relevant stimuli were sounds of these seven animals, 250 ms, 60–65 dB, 44.1 kHz, adopted from Marcell et al. (2000), presented to both ears. In both tasks, the deterministic item for categorization was a dog or a bird. As a result, the visual distractors were pictures of a dog or bird selected from the same set as the task-relevant stimuli; the auditory distractors were sounds of a dog or bird, 5,000 ms (sufficient for responding based on the results of the pilot experiment), 40–45 dB, 44.1 kHz, adopted from Marcell et al. (2000), presented to one ear randomly. It should be noted that there was no overlap between the target sounds and the auditory distractors, as they were taken from separate sections of a lengthy audio clip. Such manipulation has been employed (Murphy et al., 2013), and it was effective in making the target and distractor sounds separable and preventing them from being perceived as the same sound.

### 2.3 Experimental design

This study included two experiments. Each experiment conforms to a three-factorial design (no-distractor, ND vs. auditory distractor, AD vs. visual distractor, VD). Human categorization is considered to reflect the derivation of optimal estimates of the probability of features of objects (see review, Anderson, 1991). In empirical categorization research, the task-relevant stimuli were items with a deterministic feature and several non-deterministic or probabilistic features (Hoffman and Rehder, 2010; Deng and Sloutsky, 2015, 2016). Correspondingly, in the current work, the target frame containing six animals (i.e., six animal images in visual categorization and six animal sounds in auditory categorization) served as a categorization stimulus. The deterministic animal (i.e., dog or bird) was presented in any of the six positions in the visual space or the sound sequence, similar to the deterministic feature of a typical categorization stimulus, while the other five animals were non-deterministic. On each trial with a distractor, the visual distractor was a bird or a dog appearing above or below the black frame and the auditory distractor was the sounds of a bird or dog presented to left or right randomly. Therefore, this categorization paradigm allowed for an investigation on the impacts of visual or auditory distractors on visual and auditory categorization.

#### 2.3.1 Visual categorical decision-making task

**Figure 1:**
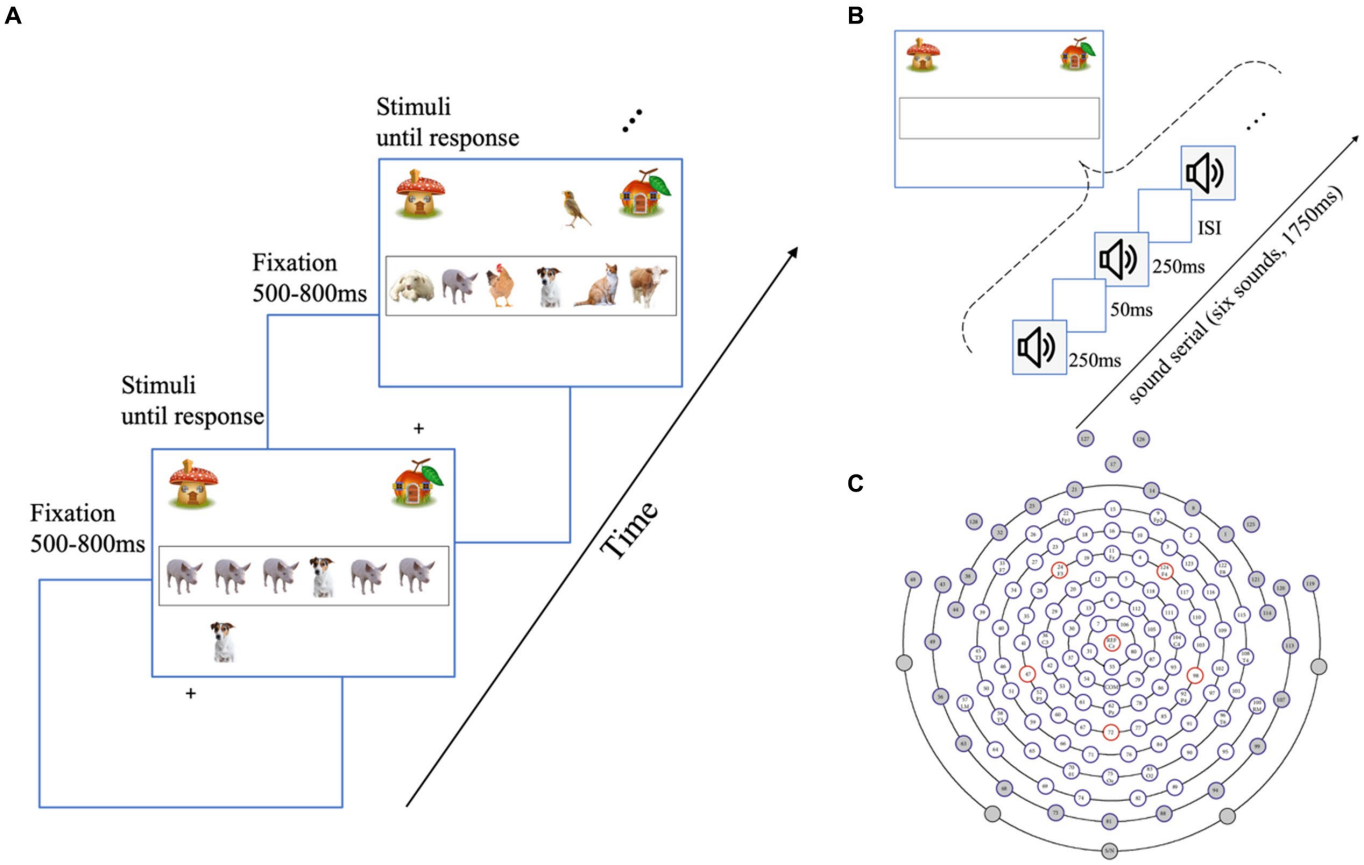
Experimental design and channel selection. **(A)** Schematic representation of the visual distractor condition of the visual task. Participants were required to categorize a set of animals in the black frame either a dog or a bird presented and indicate their choice with a keypress, followed by an inter-stimulus interval for 500–800 ms. In the no-distractor condition, only the task-relevant animals were presented, with no animals outside the black frame. In the auditory distractor condition, preserving the visual stimuli as the no-distractor condition, the sounds of a bird or dog are playing randomly in the left or right ear. **(B)** Example of the task-relevant information presentation in each trial of the auditory task. The sounds of animals were presented for 250 ms, followed by a 50 ms silence interval. The auditory task follows the identical structure as the visual task, with a 500–800 ms fixation and each trial lasting until response. Participants were instructed to press response as soon as they get the categories rather than waiting for the entire series. Visual and auditory distractors are presented in the same way as the visual task. **(C)** The 128-electrode position on the scalp. The gray colors indicate unprocessed channels. The remaining 98 channels were used for analysis.

As illustrated in Figure 1A, the task-relevant information was an array of animals presented simultaneously in the target area (i.e., inside a black frame), one deterministic item in each trial, either a dog or a bird presented at any of the six locations, and the other five positions were filled by different (cats, pigs, etc.) or identical non-target animals (e.g., probabilistic features, the five other features were the same, or they were different animals to control the diversity among trials). In the no distractor (ND) condition, there was only the target without any distractors. The task-relevant stimuli inside the black frame were presented until a response was made. Participants were instructed with a cover story:

“*Welcome to our game named Help Animals Go Home. Some animals will be presented in the black frame in the center of the screen. If there is a dog in the black frame, these animals are living in the mushroom* (see Figure 1, the top-left corner of the screen)*, and you should press the F key to help them back home. If there is a bird presented in the black frame, these animals live in the apple room* (see Figure 1, the top-right corner), *and you should help them back home by pressing the J key on the keyboard.”*

In the visual distractor (VD) condition, a bird or a dog appears above or below the black frame. Correspondingly, in the auditory distractor (AD) condition, auditory distractors were randomly presented to the left or right side. This approach, previously utilized in auditory distractor studies (Murphy et al., 2013; Robinson et al., 2018), aims to prevent the perception of the target and distractor as a combined entity, thereby generating an appropriate distractor effect. Participants were instructed to pay attention to the task-relevant information inside the frame. On each distractor trial, (1) the onset of task-relevant stimuli and distractors was the same as that of the trial, and they were presented until participants made a response; (2) these distractors could trigger either the same or different responses from the target (congruent distractor/incongruent distractor) to prevent participants from using a specific strategy of paying attention only to the distractors: if the distractor is constantly the same or different from the target, participants can optimize their task performance by ignoring the target and relying exclusively on the distractor directly related to a response. Notably, we did not treat congruency as an independent variable in our current data analysis. Instead, we controlled for it as a random factor since previous behavioral experiments using the same paradigm did not reveal an effect of congruency (Li and Deng, 2023b), which is consistent with previous research employing a similar paradigm demonstrating no congruency effect of unimodal distractors on adults (Matusz et al., 2015; Robinson et al., 2018).

#### 2.3.2 Auditory categorical decision-making task

As we stated above, considering the physical properties of auditory stimuli, the temporal separation was more appropriate for auditory stimuli. As shown in Figure 1B, each trial consisted of a rapid sequence of six animals’ sounds (Murphy et al., 2013), a target sound (dog bark or bird chirp) with other five non-target sounds. These sounds were separated by silent intervals of 50 ms, resulting in a total duration of 1750 ms for each sequence. Similar to the visual task, participants were instructed that:

“*Welcome to our game named Help Animals Go Home. A few animals are hiding behind the black frame. Please pay attention to the target sequence and send animals back home depending on whether there was a dog bark or bird chirp. If there is a dog in the sound sequence, then these animals live in the mushroom, and you should press the F key to help them back home. If there is a bird in the sound sequence, then these animals live in the apple room*, *and you should press the J key to help them back home. The bird and dog sound will not appear in the same trial*. *Please respond as soon as you make a choice, rather than waiting for the whole sound sequence.*”

Similar to the visual task, the dog or bird sound was presented randomly at any of the six positions, and the other five positions were filled by different or identical (cats, pigs, etc.) animal sounds.

For the AD and VD conditions, the distractor presentation was the same as that of the visual task. The visual or auditory distractors were presented at the onset time of the first sound in the sound serial since the onset of distractors was the same as that of the task-relevant information (the entire sound serial) in each distractor trial. And distractors would be presented until response (ending the trial). Notably, the response time of the auditory task was the duration from the onset of bird chirp or dog bark to participants’ responses.

### 2.4 Procedure

Participants sat approximately 50 cm from a laptop screen. The experiment consists of instructions, practice, EEG recording preparation, and testing.

#### 2.4.1 Instructions and practice

Before practice, participants were instructed with the cover story about two categories of animals. They read the instructions and questions on the computer screen and pressed the keyboard to make responses. After instructions, participants were given practice trials to make sure they understood the procedure. They needed to practice each condition until the accuracy rate of the last five trials was not less than 80%, and they were required to complete at least 10 trials.

#### 2.4.2 EEG recording preparation and testing

After practice, the experimenter helps participants to wear an electrode cap and an in-ear headphone. The testing phase is administered immediately after the EEG recording is prepared. For the three conditions of each task, the ND condition was always the first (to establish a baseline categorization performance), followed by the VD and AD conditions in random order. In each task, there are 60 trials in the ND condition and 120 trials in the other two distractor conditions (240 total trials). The location of the visual distractor (above/blow frame), auditory distractor (left/right ear), the variability (the probabilistic features were the same or different), and the congruency (the task-relevant information and distractors were eliciting the same or different responses) were random, and the order of test modality (AD/VD), the specific response keys for two categories, and the test order of two tasks were fully counterbalanced with each participant.

### 2.5 Data analysis

We analyzed the behavioral performance (see section 2.5.1), HDDM parameters (see section 2.5.2), and EEG signals (see section 2.5.3) of the two experiments (i.e., visual categorization and auditory categorization) separately. Since task-relevant stimuli were presented simultaneously in the visual task but sequentially in the auditory task, it’s somewhat inappropriate to compare the two tasks directly. Because the visual task and auditory task were completed in blocks, with participants finishing three conditions of each task in random order, this design allowed for separate analyses. Importantly, such analysis would serve the purpose of the current study, which was to investigate the impacts of visual and auditory distractors on visual and auditory categorical decision-making and to determine if the impact mechanisms were the same or different for each task.

**Figure A1:**
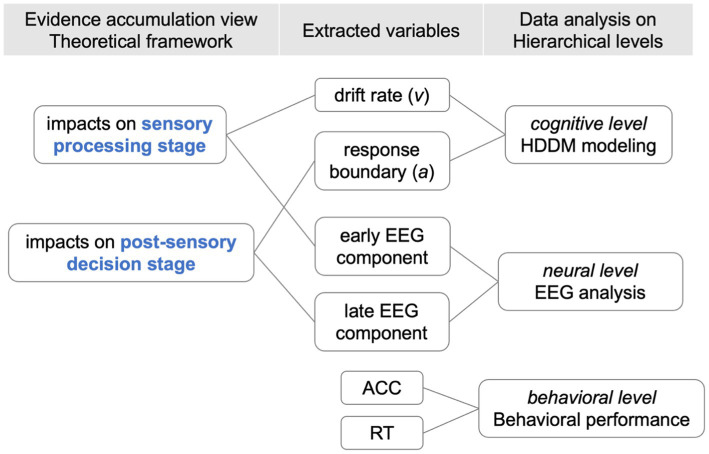
The framework diagram. The analysis outline and the key variables extracted from the behavioral, modeling, and EEG analysis.

Please refer to Appendix Figure A1 for the analysis outline and the key variables extracted from the behavioral, modeling, and EEG analysis.

#### 2.5.1 Behavioral analysis

We analyzed the visual task and auditory task separately. For each task, trials with a reaction time (RT) exceeded 3 standard deviations of that participants’ mean RT were first excluded from the analyses. To investigate how the task performance was affected by the distractors, we applied two linear mixed effect models on each task, with reaction time (RT) and response accuracy (ACC) as dependent variables, Condition (ND vs. AD vs. VD) as a fixed factor, and ‘Subject ID’ and ‘Stimuli’^1^ as random factors. These analyses were performed in RStudio (R CoreTeam, 2013). Results were analyzed by fitting linear mixed models using the *lmer* (RT data), *glmer* (ACC data), and *anova* functions in the *lme4* and *lmerTest* package in R (Bates et al., 2014; Kuznetsova et al., 2017). Post hoc tests were done with pooled t-tests by using the *emmeans* function provided by the *emmeans* package (Lenth et al., 2018).

#### 2.5.2 Hierarchical drift-diffusion modeling analysis

Participants’ performance (RT and dog-category or bird-category choice accuracy for each trial) was fitted with Hierarchical drift-diffusion modeling (HDDM). This model was used to figure out the impacts of task-irrelevant on latent cognitive processes underlying decision-making and link them to neural mechanisms (Ratcliff et al., 2016). Similar to the traditional DDM, the HDDM implies that noisy sensory evidence is sampled from the environment over time until it reaches one of two categorical choices (here, corresponding to dog-category and bird-category choices), prompting a choice decision.

The model returns estimates of internal cognitive processes (Ratcliff et al., 2016; Murray et al., 2020; Shamloo and Hélie, 2020): (1) a probable bias toward one of the two responses or an initial preference for one of them is represented by the initial bias (preference) *z*; (2) the speed of information accumulation is represented by the drift rate parameter *v*, which is attributed to the perceptual accumulation of ‘target’ evidence and donating for the sensory processing account; (3) the amount of information required to trigger response is depicted by the boundary separation *a*, representing the distance between the start point and the decision boundaries (boundary for bird-category and boundary for dog-category) and donating for the post-sensory decision account; (4) time not related to decision process (for example, the time required for sensory information encoding preparation or the time for motor response execution) is represented by non-decision time parameter *t*. The difference in this parameter is not due to the evidence accumulation processes.

A hierarchical Bayesian framework (updating prior model parameter distributions based on the likelihood of the data) was used to calculate the modeling parameters and then get the posterior distributions. To maximize the summed log-likelihood of the predicted data, Markov chain Monte Carlo (MCMC) sampling was utilized (Wiecki et al., 2013; Wabersich and Vandekerckhove, 2014) with 5,500 samples chosen from the posterior to generate smooth parameter estimates, and the first 500 samples were discarded as burn-in. Besides, to check that the models had appropriately converged, the autocorrelation and Gelman–Ruben statistic were computed after inspecting traces of model parameters (Gelman and Rubin, 1992). Compared to the traditional DDM analysis, the current modeling has significant advantages. Foremostly, HDDM analysis is useful for enhancing statistical power. Secondly, the uncertainty of parameter estimates can be conveyed directly by posterior distributions (Kruschke, 2010). Thirdly, the Bayesian hierarchy helps with more reliable results even though few trials (Ratcliff and Childers, 2015). Lastly, all observers are from the same group in this hierarchical structure, resulting in more reliable parameter estimations for individual participants (Wiecki et al., 2013).

To determine the parameters, the HDDM was implemented in Python 3.9.^2^ According to the hierarchical design, individual parameters are generated and constrained by group distributions with group priors (Gelman et al., 2013). Parameters were drawn from group-level Gaussian distributions. Trials that fell within 5% of each tail of the RT distribution were considered outliers (e.g., slow replies as a result of inaction or quick but incorrect reactions) and removed from the analysis (Wiecki et al., 2013).

We estimated HDDM models based on the behavioral performance of the visual task and auditory task separately. With the assumption that no response bias to two categories (dog-related-category and bird-related-category), to discover the best model, eight distinct models were evaluated in both tasks, with three parameters of interest (*v*, *a, t*) either fixed or allowed to vary on conditions across the eight model variants (model 1: all three parameters were fixed; model 2: *v* was varying; model 3: *a* was varying; model 4: *t* was varying; model 5: *a* and *t* were varying; model 6: *v* and *a* were varying; model 7: *v* and *t* were varying; model 8: *v*, *a* and *t* were varying between conditions). To compare the models, the deviance information criterion (DIC), which is the lower, the better (Zhang and Rowe, 2014), reflecting the best trade-off between model complexity and quality of fit, was used to select the best model to describe the data across the eight models (Spiegelhalter et al., 2002). Lower DIC values favor models with the highest likelihood and least degrees of freedom. A DIC difference of 10 is considered significant, and the lower the value, the better the model fit (Zhang and Rowe, 2014). In addition, after getting the best-fitting model, we examined the between-group overlap of the Bayesian posterior distributions for all parameters, defining significance as less than 5% overlap, to show the difference between no-distractor and distractor conditions.

#### 2.5.3 EEG data analysis

Briefly, we first preprocess the EEG data. Secondly, to estimate linear spatial weights, which maximally represented the difference representations between no-distractor and distractor contexts within short predefined temporal windows, the single-trial multivariate discriminant analysis was performed for each participant. By applying the spatial weights of single-trial data, an index of the extent of the discrimination on representations, and the discriminating component amplitudes (henceforth *y*) were calculated. The more extreme amplitudes, positive or negative, indicate higher representation discrimination between no-distractor and distractor contexts, while values closer to zero indicate no evidence of encoding differently. Thirdly, we employed a *k*-means clustering technique to detect temporal transitions to identify EEG components. And finally, rather than a guess probability, we determined the criterion for each comparison using the temporal cluster-based bootstrap analysis.

##### 2.5.3.1 EEG data acquisition and preprocessing

The task was presented on a Dell laptop (1,024 × 768 pixels, 60 Hz) using the Eprime 2.0 with recoding experimental events and behavioral responses. Continuous EEG data were collected in a sound-attenuated and electrostatically insulated environment utilizing 128-channel HydroCel Geodesic Sensor Nets coupled to Net Amps 300 (Electrical Geodesics) and NetStation software. EEG data were acquired at a sampling rate of 1,000 Hz with an analog filter of 0.01–100 Hz and the electrode impedances of all channels were adjusted below 50 kΩ. These data were gathered and saved for offline analysis with MATLAB.

All EEG data preprocessing was carried out offline with the EEGlab toolbox (v2021.0) (Delorme and Makeig, 2004) of MATLAB R2017b. Firstly, 98 out of 128 channels were selected (Figure 1C, gray channels deleted and white channels remained for analysis), discarding all channels outside the scalp (Luu and Ferree, 2005), corresponding to the previous research, in which 64-channel EEG was used and dynamic temporal components of face-vs.-car categorization were demonstrated (Franzen et al., 2020). Secondly, all signals were re-referenced to the average of the channels, a software-based fourth-order butterworth band-pass was then filtered with cutoff frequencies between 0.5 and 40 Hz. Thirdly, automatic channel rejection and visual screening for high amplitude due to body movements, sweating, and temporary electrode malfunction were conducted, with a result that no more than 5% bad channels per participant were interpolated. In addition, to obtain event-related components, stimulus-locked epochs of 1,200 ms (200 ms pre-stimulus) were extracted from continuous data, and then Independent Component Analysis (ICA, option: runica) procedure was used to de-noise (Delorme et al., 2007). An *ADJUST* automatic classification algorithm (EEGlab plugin) (Delorme et al., 2007) and manual screening on topographical distribution and frequency contents were employed to reject components that reflected eye movement-induced muscle activity. The EEG signal that exceeded ±120 μV in amplitude during the epochs was then deleted.

##### 2.5.3.2 EEG single-trial discrimination analysis

To identify (1) the effect of task-irrelevant information in each sense; (2) the dynamic time process of the effect, that is, whether the early sensory processing of information or the efficacy of post-sensory processes was impacted (Parra et al., 2005; Franzen et al., 2020), linear multivariate single-trial discrimination analysis was conducted separately for the visual and auditory task.

Specifically, we identified a projection of the multidimensional EEG data, *x_i_*, where *i* = [1, 2…*N* trials], within short sliding windows that maximally discriminated between distractor and no-distractor trials. As a result, VD-ND and AD-ND comparisons were carried out in the visual and auditory tasks, respectively. All time windows had a width of 60 ms with 10 ms onset intervals and the window center was shifted from −100 to 900 ms relative to stimulus onset. In particular, logistic regression was used to learn a 98-channel spatial weighting, 𝜏, that achieved maximal discrimination within each time window, arriving at the one-dimensional projection *y_i_* (*τ*), for each trial i and a given window 𝜏:


(1) $$y\left(\tau \right)=w{\left(\tau \right)}^{T}x\left(\tau \right)=\overset{D}{\underset{i=1}{\sum }}w_{i}\left(\tau \right)x_{i}\left(\tau \right)\text{,}$$

where *T* refers to the transpose operator and *D* stands for the number of EEG sensors. The discriminator was built to convert component amplitudes *y_i_* (*τ*) to positive and negative values for no-distractor and distractor trials, which are the weighted reflection of neural evidence of the difference between no-distractor and distractor contexts. A larger positive or negative value indicates a higher possibility of a no-distractor or distractor context, whereas values approaching zero imply difficulty in differentiating context.

We employed the area under a ROC curve (Green and Swets, 1966), referred to as an *Az* value, together with a leave-one-trial-out cross-validation approach to compensate for overfitting and measure the performance of our discriminator for each time frame (Philiastides and Sajda, 2006a,b, 2007; Philiastides et al., 2006). Specifically, we used N-1 trials to estimate a spatial filter *w*, which was then applied to the left-out trial to extract out-of-sample discriminant component amplitudes (*y*) and compute the *Az* value for each iteration. Furthermore, we used a bootstrap approach (see *temporal cluster-based bootstrap analysis*), as well as a separate leave-one-trial-out test, to access significance thresholds for discriminator performance rather than assuming an *Az* = 0.5 as chance performance. This procedure was done 1,000 times to produce a probability distribution for *Az*, which was utilized as a reference to estimate the *Az* value, resulting in a significance level of *p* < 0.05. This EEG analysis pipeline was run on separate participants, making each one their own replication unit (Smith and Little, 2018).

Finally, given the linearity of our model, we computed the scalp projections of our discriminating components arising from Eq. (1) by estimating a forward model as follows:


(2) $$a\left(\tau \right)=\frac{x\left(\tau \right)y\left(\tau \right)}{y{\left(\tau \right)}^{T}y\left(\tau \right)}\text{,}$$

where the EEG data (*x*) is in a matrix and discriminating components (*y*) are in vector notation. These forward models can be shown as scalp plots and interpreted as showing the normalized correlation between the discriminant output and the EEG activity (Philiastides et al., 2014). In both the visual and auditory tasks, these forward models were estimated separately for AD-ND comparison and VD-ND comparison.

##### 2.5.3.3 Optimizing the number of distinct spatiotemporal components

During periods of persistent significant discriminating activity, we employed the forward model estimates from Eq. (2) to identify temporal transitions between various components based on scalp distribution discrepancies, which are suggestive of changes in the underlying cortical sources. Specifically, we used a *k*-means clustering technique with a Euclidean distance metric on the intensities of vector *a*(*τ*) for the entire time range of interest and optimized *k* (i.e., the number of different time windows with similar scalp topographies) using *silhouette* values (Rousseeuw, 1987), as implemented in MATLAB’s *evalclusters* function. Moreover, regardless of the criterion employed, the distance metric used for clustering, and the settings for both comparisons, our results were consistent. The obtained temporal components were used in all relevant EEG analyses.

##### 2.5.3.4 Temporal cluster-based bootstrap analysis

The primary goal of this study is to figure out when the categorical decision is influenced by task-irrelevant information and then to discuss if visual and auditory distractors have an impact on different stages of evidence accumulation in the visual and auditory tasks. As a result, we attempted to identify temporal windows during which the discriminator performance of the AD-ND and VD-ND comparisons differed. Then figure out when it intersects with the early and/or late components. We utilized a percentile bootstrap approach to compare the group-level *Az* difference between two dependent samples to quantify whether and when the discriminator performance changed between the VD-ND and AD-ND comparison (Rousselet et al., 2016). Specifically, we produced a distribution of shuffled *Az* difference scores (AD-ND comparison – VD-ND comparison) among individuals on a sample-by-sample basis. For each sample, we repeated the shuffling method 1,000 times, resulting in a random bootstrap distribution with the median *Az* difference scores from each iteration. In addition, we calculated the average of this bootstrap distribution and evaluated the difference of bootstrapped mean against the lower bound of the calculated confidence interval (*p* < 0.025, at the 2.5% threshold) to see if it was statistically different from zero.

A minimum of three significant samples was required to form contiguous temporal clusters and prevent transient effects caused by false positives. The 95th percentile of a data-driven null distribution of maximum cluster sizes was used to calculate this criterion. While the relationship between adjacent samples was preserved in the previous analysis, we used a permutation procedure (shuffling temporal samples without replacement) to abolish the relationship between temporal samples, while keeping the relative difference between AD-ND and VD-ND comparison *Az* values for each sample and participant unchanged. We computed and stored the maximum number of adjacent significant samples of the largest cluster for each of the 1,000 repetitions to construct the null distribution of maximum cluster sizes. This analysis was performed on the discriminator performance (*Az*), which generated at least two significant samples in each comparison (it is also possible that both components are significant in VD-ND and AD-ND comparison). This technique accounts for multiple comparisons and is analogous to the temporal cluster-based non-parametric permutation test (Maris and Oostenveld, 2007).

Furthermore, the proportion of individuals who exhibited a participant-level impact in line with the general group-level effect was calculated to ensure that neural effects could be consistently traced in individual participants without group-level averages obscuring variability. These statistical analyses were carried out using MATLAB code (Rousselet et al., 2016, 2017).

## 3 Results

### 3.1 Visual task: visual and auditory distractors exhibited differential effects on the neural level

**Figure 2:**
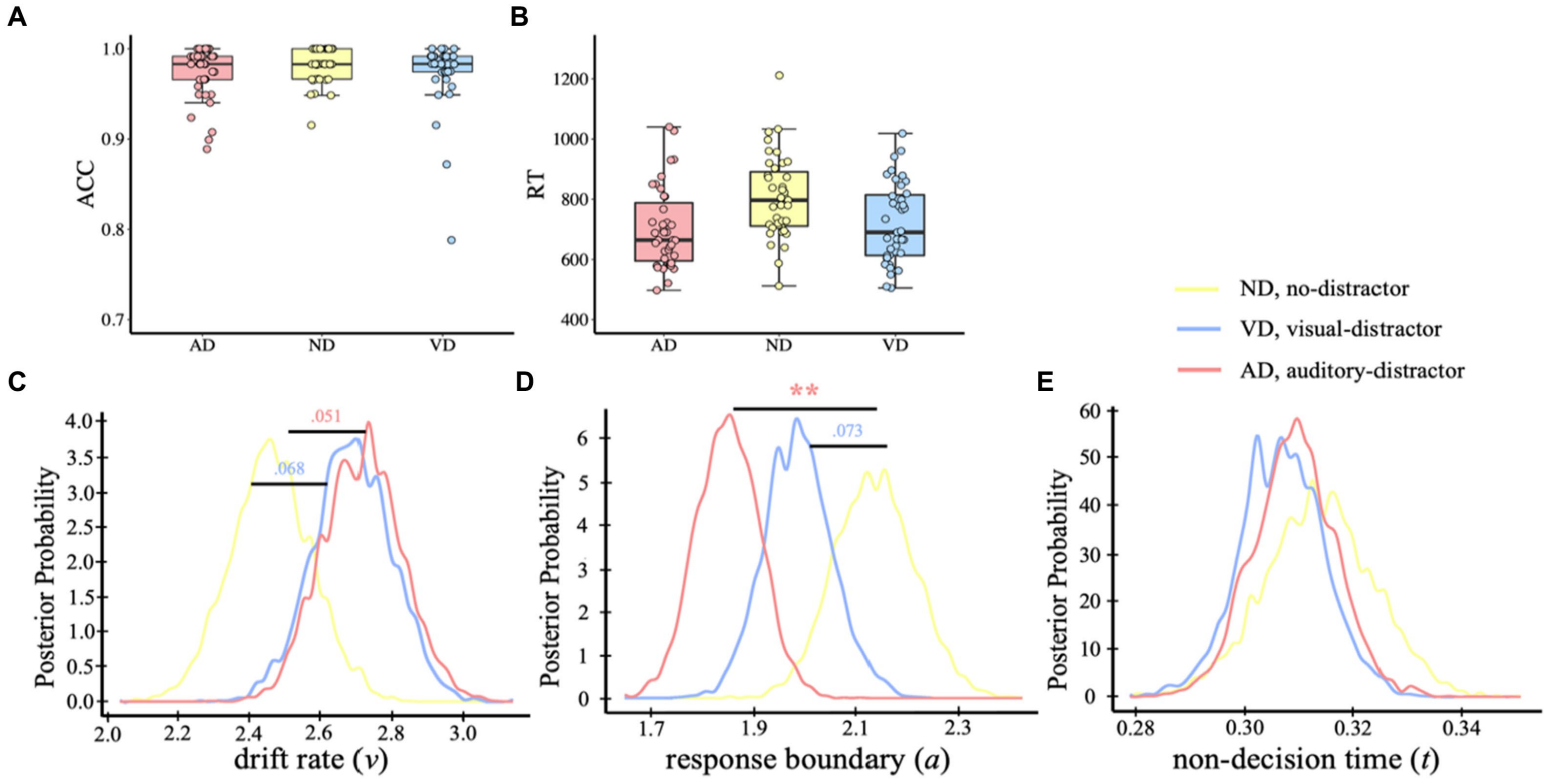
Behavior analysis and HDDM results of visual task. ACC **(A)** and RT **(B)** of the visual categorical decision-making task. Dots indicate single-participant values for each condition. Plots of posterior probabilities for HDDM parameters: drift rate *v* **(C)**, response boundary *a* **(D)**, non-decision time *t* **(E)**. **p < 0.01.

To assess the effect of intermodal and intramodal distractors on the visual evidence accumulation process, we used two linear mixed-effects models with random intercepts for the subject and stimuli to examine the effects of distractor condition (AD vs. ND vs. VD) on ACC and RT. Participants responded within the time limit in all trials. We excluded trials with reaction times that deviated more than 3 standard deviations from the mean. In the analysis, a small percentage of trials from each condition were excluded: 1.90% from the AD condition, 2.39% from the ND condition, and 1.73% from the VD condition. For both ACC (Figure 2A) and RT (Figure 2B), no difference was found between no-distractor and distractor conditions (*ps* > 0.05; ACC: *M*_AD_ = 0.973, *SD*_AD_ = 0.029; *M*_ND_ = 0.981, *SD*_ND_ = 0.019; *M*_VD_ = 0.974, *SD*_VD_ = 0.039; RT: *M*_AD_ = 703 ms, *SD*_AD_ = 134 ms; *M*_ND_ = 806 ms, *SD*_ND_ = 137 ms; *M*_VD_ = 721 ms, *SD*_VD_ = 132 ms).

To determine which functional level the distractor affects in the visual task, we compared multiple hypothetical hierarchical drift-diffusion models, which were used to figure out which cognitive processes the task-irrelevant information was affecting. Specifically, we examined the best model that fitted the behavioral data first and then compared the parameters (drift rate, *v*; decision boundary, *a*; non-decision time, *t*) among conditions. We built eight models and the model comparison showed that model 8, with all three parameters free to vary, was the best-fit model, as a lower value of the variance information criterion (DIC) indicates a better balance between complexity and model fit (DIC_model 8_ = −1880; DIC_model 1_ = −1,249; DIC_model 2_ = −1734; DIC_model 3_ = −1728; DIC_model 4_ = −1,657; DIC_model 5_ = −1816; DIC_model 6_ = −1825; DIC_model 7_ = −1862). In addition, model 8 demonstrated excellent convergence for all estimated parameters: Rubin statistic values, Mean*_v_* ± SD*_v_* = 1.00102 ± 0.00156, range = [0.99996, 1.00877]; Mean*_a_* ± SD*_a_* = 1.00164 ± 0.00209, range = [1.00002, 1.01220]; Mean*_t_* ± SD*_t_* = 1.00138 ± 0.00186, range = [0.99994, 1.01097] (Rubin statistic values <1.1 are considered to indicate acceptable convergence). The results of model 8 revealed no initial preference for a dog-category or a bird-category response *p*_Bayes_(bias >0.50) = 0.179. There was moderate evidence for a difference in drift rate [Figure 2C, *p*_Bayes_(ND > VD) = 0.068; *p*_Bayes_(ND > AD) = 0.051], indicating higher evidence accumulation rate when with either a visual or an auditory distractor. In addition, the response boundary was lower in either visual or auditory distractor condition, compared with no distractor condition [Figure 2D, *p*_Bayes_(ND < VD) = 0.073; *p*_Bayes_(ND < AD) = 0.001], indicating that responses were more cautious when with a distractor. No evidence for a non-decision time difference between no-distractor and distractor conditions [Figure 2E, *p*_Bayes_(ND > VD) = 0.715; *p*_Bayes_(ND > AD) = 0.652].

Next, we sought to identify the early (sensory processing) and late (post-sensory decision) EEG components that discriminate the no-distractor and distractor contexts and to investigate how they are modulated by the modality of irrelevant input. A single-trial multivariate discriminant analysis was performed on visual task EEG data firstly, by comparing ND with AD and VD trials separately, to (1) detect the impact of extra task-irrelevant evidence, in other words, to identify the temporally distinct components that discriminate the visual categorical representation under no-distractor and distractor contexts; (2) characterize the magnitude of the visual and auditory distractors’ impact on visual categorical decision making.

**Figure 3:**
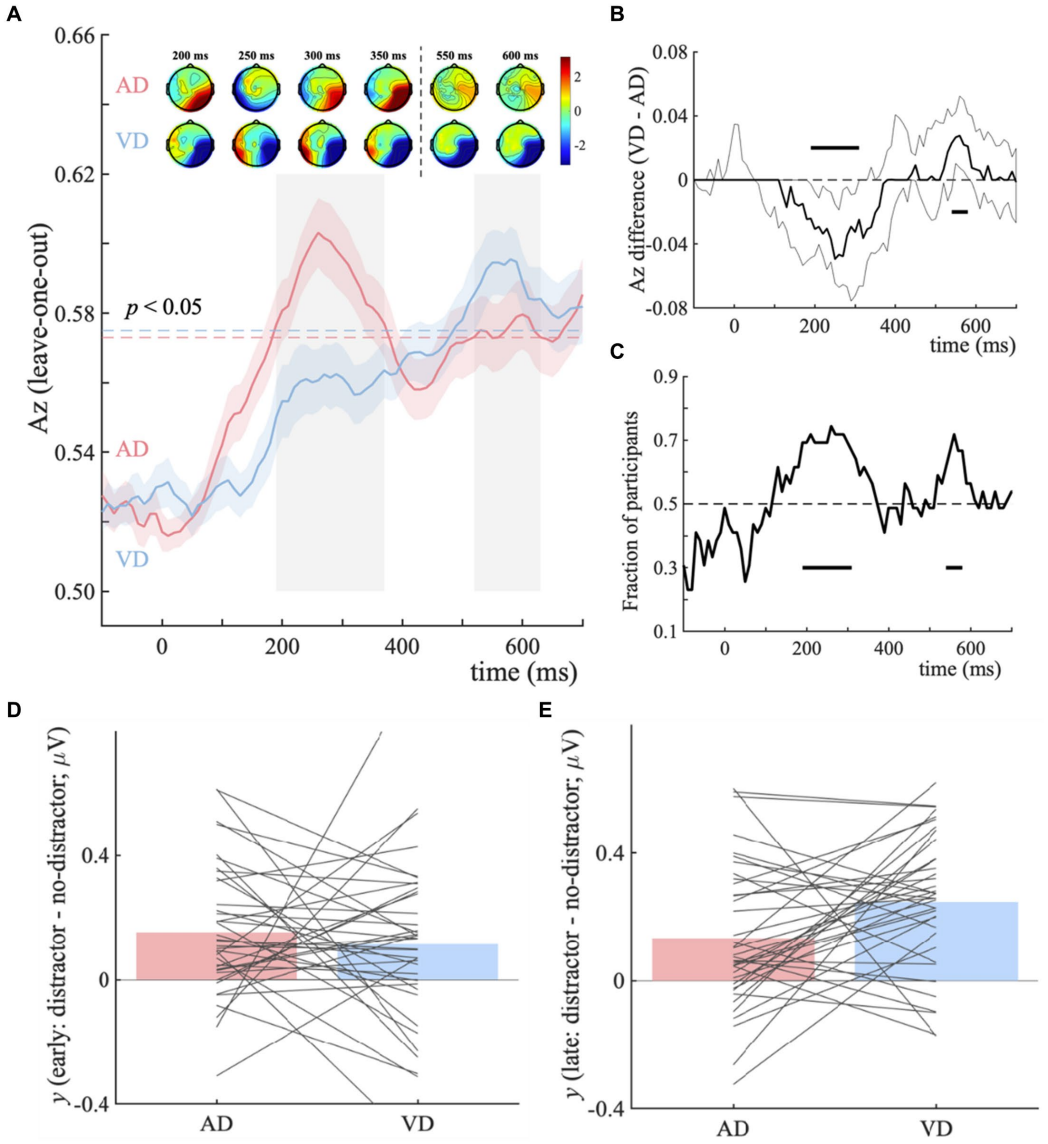
Distractor – no-distractor context discrimination analysis for visual categorical decision-making task. **(A)** Mean discriminator performance (Az) after a leave-one-trial-out cross-validation procedure for the AD-ND comparison (AD; red) and VD-ND comparison (VD; blue). Horizontal dotted lines represent the group average permutation threshold for statistical significance at *p* < 0.05 (for AD-ND comparison, Az sig = 0.573, and for VD-ND comparison, Az sig = 0.575). Bootstrapped standard errors among participants are represented by shaded error bars. To obtain the number of clusters and their extent, a *k*-means clustering algorithm with a Euclidean distance metric and optimized *k* (*k* = 2 in both VD-ND and AD-ND comparison) was used. Early (190–370 ms) and late (520–630 ms) EEG component windows computed via temporal clustering of scalp topographies are indicated by shaded gray vertical bars. These bars do not indicate statistical significance. The scalp topographies at representative time windows are used to show forward models, corresponding to the early and late EEG components. The color bar represents normalized correlation. **(B)** Bootstrapped discriminator performance difference (thick black line; VD-AD: VD-ND comparison minus AD-ND comparison) with 95% confidence intervals (thin black lines; 2.5–97.5%). Significant temporal windows (i.e., the lower confidence interval is higher than zero at *p* < 0.05, with an extra data-driven minimum criterion of three contiguous windows to correct for multiple comparisons) are illustrated by a horizontal thick black line above and below the *x*-axis. **(C)** The percentage of individuals whose discriminator performance (Az) is in the same direction as the group average. Histogram represents the early **(D)** and late **(E)** EEG component amplitudes at the point of maximal Az the separation between distractor and no-distractor trials (*y_distractor_* – *y_no-distractor_*).

For the AD-ND comparison, the discriminator’s performance reveals a broad window (190–370 ms and 520–630 ms post-stimulus, see Figure 3A, blue horizontal dotted line), indicating the categorical information represented differently. And for the VD-ND comparison, it is 490–700 ms post-stimulus (see Figure 3A, red horizontal dotted line). We used temporal clustering with a time window of 190–700 ms to determine the number of relevant components. With a transition point of 400 ms, this method reveals the presence of two temporally different scalp representations. As a result, considering the overlap with the broad window as much as possible, 190–370 ms and 520–630 ms were selected as early and late components. We next extracted participant-specific component latencies by finding the time points within the early and late component time-windows that led to peak *Az* performance, for each comparison.

The results of temporal cluster-based permutation analysis showed that temporal clusters overlapping with the early (190–310 ms) and late (540–580 ms) components (Figure 3A). For the early component, the discriminator performance of the AD-ND comparison is significantly higher, while it’s the VD-ND comparison higher for the late component (Figure 3B). Up to 74.4% of participants exhibited an early component difference and 71.8% showed a late component difference in the discriminator’s performance (Figure 3C). These results showed that the extra task-irrelevant auditory information in our task improves the quality of visual categorical evidence (as indicated by our discriminator component amplitudes *y*) during sensory processing (Figure 3D). However, the task-irrelevant visual information improves the late component primarily (Figure 3E), representing the post-sensory decision process. In addition, for AD-ND comparison, the corresponding scalp models (Figure 3A, topographical inserts, top row) revealed the strongest effects originated over occipital and temporal electrodes for the early component. For the VD-ND comparison, the early component’s topography was quite similar to that of the AD-ND comparison, but of opposite signs. For the topographies of the late component, only the VD-ND comparison (Figure 3A, topographical inserts, bottom row) showed the strongest correlations between discriminant output (*y*) and the EEG activity over posterior, temporal, and occipital regions.

These results suggested modality-specific impacts of irrelevant inputs in the visual categorical decision-making task. Specifically, the distinct effects of visual and auditory distractors showed on the neural components, with auditory distractors changing the sensory processing stage whereas visual distractors influencing the post-sensory decision stage. The impact on behavioral performance was minimal, however, the presence of both visual and auditory irrelevant inputs led to an increase in drift rate and a decrease in response boundary.

### 3.2 Auditory task: visual and auditory distractors exhibited differential effects on the behavioral and modeling-oriented cognitive level

**Figure 4:**
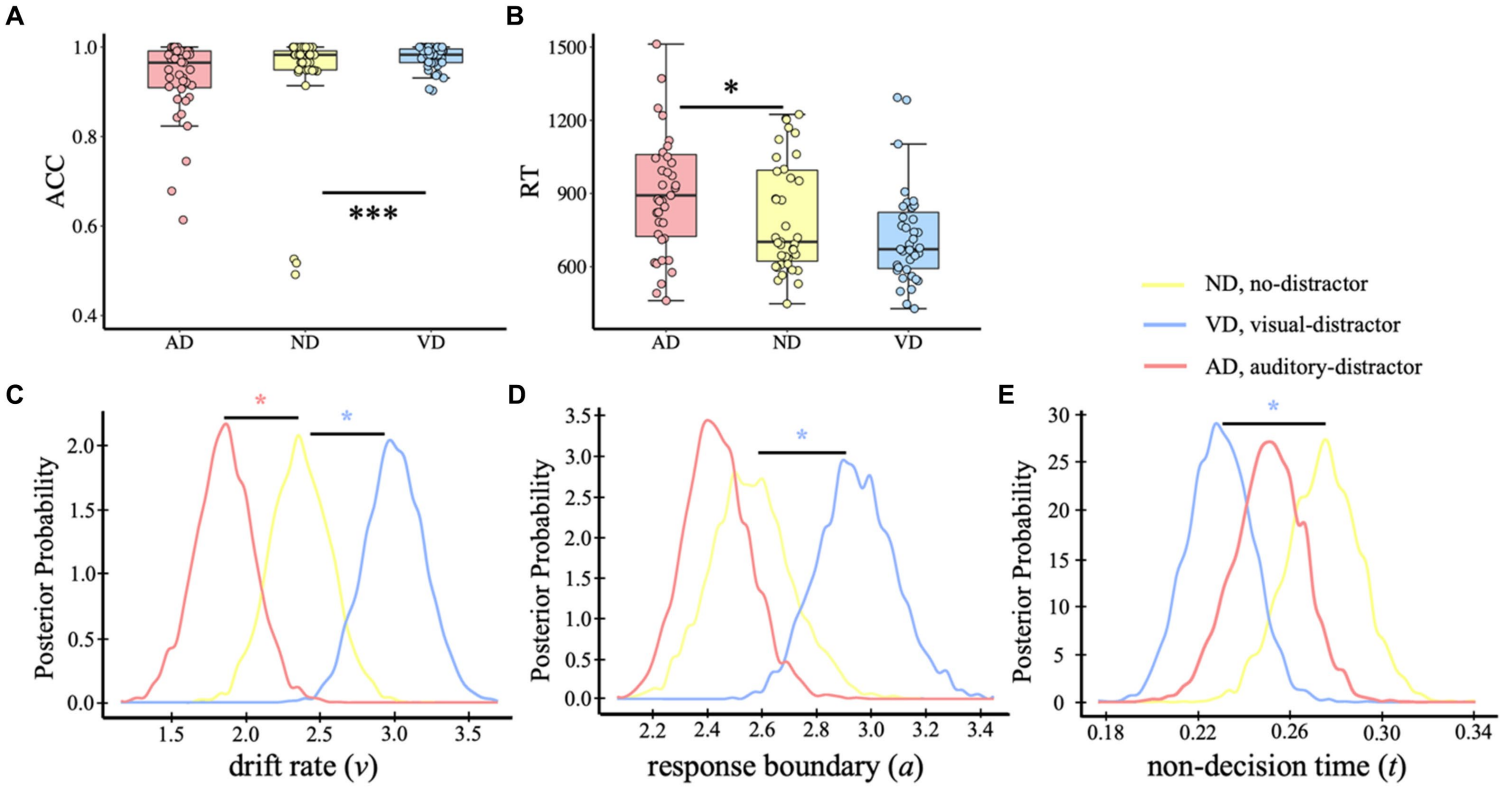
Behavior analysis and HDDM results of the auditory task. ACC **(A)** and RT **(B)** of the auditory categorical decision-making task. Dots indicate single-participant values for each condition. Plots of posterior probabilities for HDDM parameters: drift rate *v* **(C)**, response boundary *a* **(D)**, non-decision time *t* **(E)**. *p < 0.05, ***p < 0.001.

We first removed the trials in which the participants responded before the target presentation, and then deleted trials with reaction times that fell outside of three standard deviations. In the analysis, a small percentage of trials from each condition were excluded: 4.55% from the AD condition, 3.03% from the ND condition, and 3.08% from the VD condition. Same to the analysis on the visual task, in the auditory categorical decision-making, analysis on ACC (Figure 4A) showed different impacts of visual and auditory distractors, showing that auditory categorical performance was facilitated by visual distractors (*p* < 0.001) whereas auditory distractors had no effects (*p* = 0.687; *M*_AD_ = 0.929, *SD*_AD_ = 0.089; *M*_ND_ = 0.941, *SD*_AD_ = 0.127; *M*_VD_ = 0.977, *SD*_VD_ = 0.026). For RT (Figure 4B), auditory task performance was slowed down by the auditory distractors (*p* = 0.015) but wasn’t affected by the visual distractors (*p* = 0.344; *M*_AD_ = 960 ms, *SD*_VD_ = 359 ms; *M*_ND_ = 818 ms, *SD*_ND_ = 258 ms; *M*_VD_ = 742 ms, *SD*_VD_ = 247 ms).

The results of HDDM revealed that model 8, in which all three parameters were free to vary, was the best-fit model (DIC_model 8_ = 6,421; DIC_model 1_ = 6,544; DIC_model 2_ = 6,642; DIC_model 3_ = 7,313; DIC_model 4_ = 7,702; DIC_model 5_ = 7,572; DIC_model 6_ = 6,859; DIC_model 7_ = 8,440). In addition, model 8 demonstrated excellent convergence for all estimated parameters: Rubin statistic values, Mean*_v_* ± SD*_v_* = 1.00088 ± 0.00143, range = [0.99992, 1.00602]; Mean*_a_* ± SD*_a_* = 1.00160 ± 0.00229, range = [1.00002, 1.01008]; Mean*_t_* ± SD*_t_* = 1.00144 ± 0.00221, range = [1.00002, 1.00986]. The posterior distributions of model 8 revealed no initial preference for a dog-category or a bird-category response, *p*_Bayes_(bias >0.50) = 0.233. It was a higher drift rate when with visual distractors but lower when with auditory distractors [Figure 4C, *p*_Bayes_(ND > VD) = 0.016; *p*_Bayes_(ND < AD) = 0.037]. In addition, compared with the no-distractor condition, the visual distractors resulted in a wider response boundary, but no significant impact of auditory distractors [Figure 4D, *p*_Bayes_(ND > VD) = 0.019; *p*_Bayes_(ND > AD) = 0.77]. The non-decision time of the visual distractor condition was shorter than no-distractor condition but no evidence for the difference between no-distractor and auditory distractor condition [Figure 4E, *p*_Bayes_(ND < VD) = 0.019; *p*_Bayes_(ND > AD) = 0.865].

**Figure 5:**
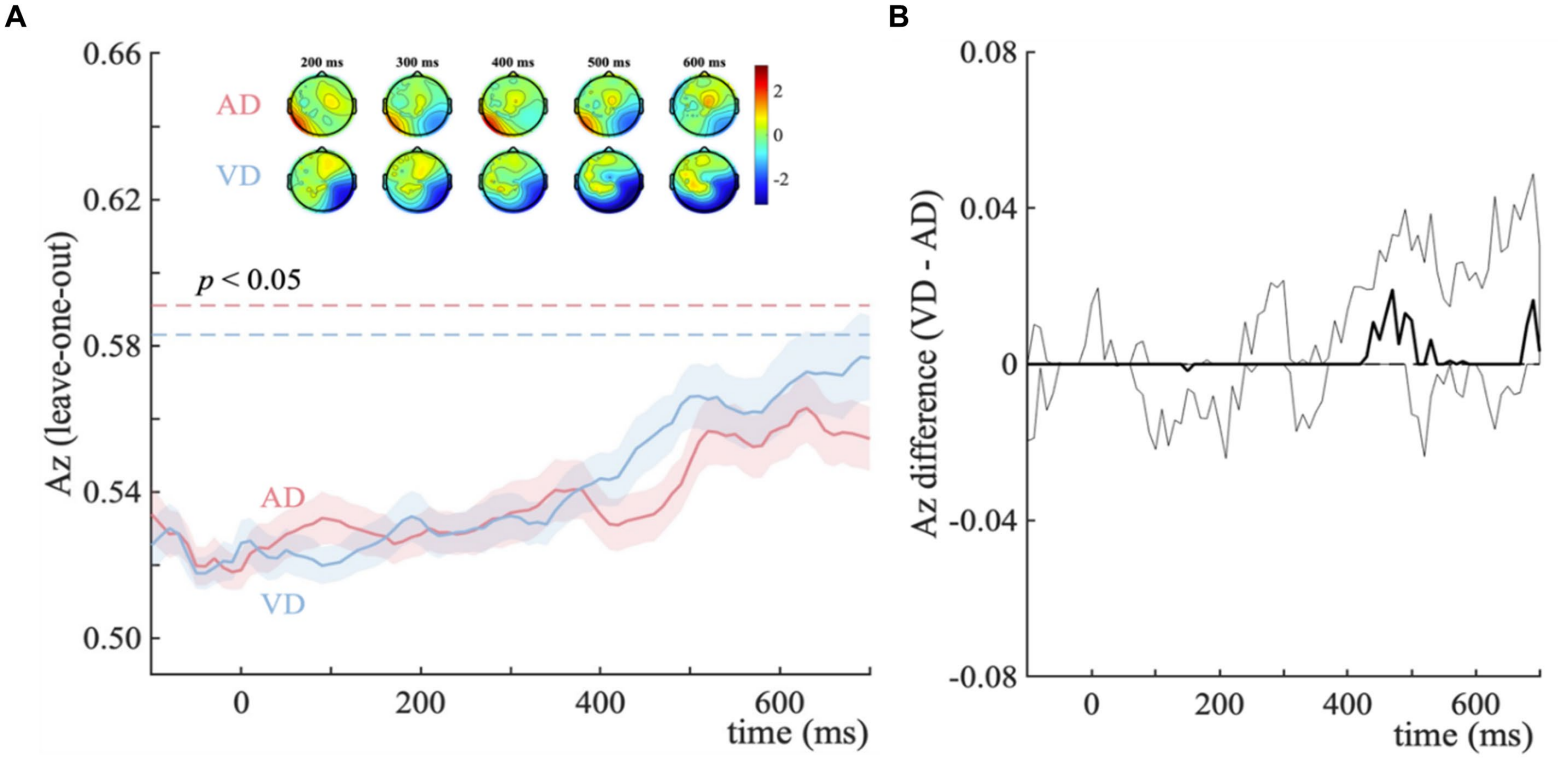
Distractor – no-distractor context discrimination analysis for auditory categorical decision-making task. **(A)** Mean discriminator performance (Az) after a leave-one-trial-out cross-validation procedure for the AD-ND comparison (AD; red) and VD-ND comparison (VD; blue). Bootstrapped standard errors among participants are represented by shaded error bars. Horizontal dotted lines represent the group average permutation threshold at *p* < 0.05 (for AD-ND comparison, it was Az sig = 0.591, and for VD-ND comparison, Az sig = 0.583). Scalp topographies display the forward models at 200–600 ms. The color bar shows a normalized correlation. **(B)** Bootstrapped discriminator performance difference (thick black line; VD-AD: VD-ND comparison minus AD-ND comparison) with 95% confidence intervals (thin black lines; 2.5–97.5%).

In the analysis for the auditory categorical information representation, we used the same analysis method as the visual categorical decision-making. We compared the different effects of additional task-irrelevant visual and auditory information. No significant discriminator performance showed in both the VD-ND and AD-ND comparisons (Figures 5A,B). That is, we did not find the temporally distinct components between no-distractor and distractor contexts in the auditory task.

Collectively, we observed modality-specific impacts of irrelevant inputs in auditory categorical decision-making, which is consistent with the findings of the visual task. In the auditory task, the distinct effects of visual and auditory distractors showed on the behavioral level and cognitive processes, showing (1) the facilitation of visual distractors but interference of auditory distractors on behavioral performance; (2) visual distractors resulting in a higher evidence accumulation rate and response boundary, indicating the impacts on both the sensory processing and post-sensory decision stages, whereas auditory distractors led to a lower evidence accumulation rate without affecting the response boundary, indicating the impacts on the sensory processing rather than the post-sensory decision stage. The impacts of irrelevant inputs were not observed on the neural level in the categorical decision-making task.

## 4 Discussion

We continually categorize information based on the features of inputs from multiple senses, yet not all sensory inputs are equally relevant. In this work, we provided evidence on how intermodal and intramodal distractors affect categorical decision-making processes from the behavioral level, cognitive level (HDDM framework), and neural level. In the visual categorical decision-making, the distinct effects of visual and auditory distractors showed on the neural component, with visual distractors changing the post-sensory decision stage whereas auditory distractors affecting the sensory processing stage. Cognitive processes were identically impacted by visual and auditory distractors, with no effect on behavioral performance. In the auditory categorical decision-making, the distinct effects of visual and auditory distractors showed on the behavioral level and cognitive processes, showing the facilitation effect of visual distractors and impacts on both the sensory processing and post-sensory decision stages, but the interference of auditory distractors and impacts on the sensory processing rather than the post-sensory decision stage. In addition, the impacts of irrelevant inputs on the auditory task were not observed on the neural level.

Therefore, the modality-specific impacts of irrelevant information on both the visual and auditory categorical decision-making were demonstrated, with the effect of irrelevant information modulated by its modality and occurring at both sensory processing and post-sensory decision stages. In the visual task, even though the behavioral performance and modeling parameters were hardly affected, they had distinct effects on the neural level. Auditory distractors resulted in an early EEG component, reflecting the effects on the perceptual evidence accumulation (Chen et al., 2021), whereas visual distractors resulted in a late component, indexing the impacts on the post-sensory decision stage (Philiastides and Sajda, 2006a,b, 2007; Philiastides et al., 2006; Ratcliff et al., 2009; Franzen et al., 2020). However, in the auditory categorical decision-making, there were no dynamic neural components, but there were distinct effects of distractors on the behavioral performance and the underlying cognitive processes, implying that visual distractors are likely to facilitate performance, possibly due to the higher evidence accumulation rate and more conservative response criterion, whereas auditory distractors tend to interfere, due to the lower evidence accumulation rate.

In this study, we were particularly interested in the effect of task-irrelevant information and the moderating role of its modality. In the field of information processing, perceptual processing and response selection are required (Halford et al., 2010). The early processing hypothesis posits that evidence from multiple modalities is combined at the sensory encoding stage and may be reflected in the early component of neural activity in primary cortices (Schroeder and Foxe, 2005; Kayser and Logothetis, 2007), whereas the late processing hypothesis states that evidence from each sensory modality is isolated originally and processed independently and supports for a response later (i.e., post-sensory decision process). However, most previous studies exploring the dynamic processes of information processing have focused on simple perceptual decision-making, such as visual detection and visual feature discrimination (Giard and Peronnet, 1999; Sajda et al., 2009; Kayser et al., 2017). It may be challenging to generalize inferences drawn from these studies to auditory decision-making or more complex cognitive tasks such as categorization.

In the visual task, our neurological results support the modality-specific early and late processing hypotheses. The early EEG component in the auditory distractor condition indicates that the representation of auditory task-irrelevant information emerges at a sensory processing stage, suggesting a perceptual origin rather than post-sensory decision process (Zhang and Rowe, 2014). Besides, it is the primary cortical regions that are involved in this process (Cappe et al., 2009; Mercier et al., 2013). These results support the sensory processing hypothesis for the processing of auditory distractors in visual categorical decision-making.

In contrast, the processing of visual distractors in the visual task showed a late neural component, supporting the late-processing hypothesis. In categorization tasks, sensory regions interact with higher-order areas, such as the parietal and frontal associative cortex and lateral occipital cortex (Philiastides and Sajda, 2007; Kayser et al., 2017; Mercier and Cappe, 2020). Visual distractors may have an effect in this manner. Specifically, all inputs are encoded separately and then combined into a single source of evidence for decision formation in the higher-order areas (Heekeren et al., 2004; Pisauro et al., 2017).

An alternative explanation is that evidence from different modalities accumulates concurrently, whereas in one modality it is sequential. As such, simultaneously presented auditory and visual information can affect sensory processing (Widmann et al., 2004; Foxe and Schroeder, 2005), and thus evidence accumulates at a higher rate, generating greater neural activity in the early stage of stimulus onset. However, with top-down control, the relevance and reliability of the information are considered, and the visual-irrelevant information is processed later.

Another possibility is that the information accumulation could be modulated by the physical properties of stimuli. A cognitive process that determines whether the received information is a target or a distractor can help individuals to select and focus on more useful information. Given that most auditory inputs are alert, dynamic, and transient, they could be prioritized so that individuals have little time to think and instead use all the information available to them to respond. In contrast, individuals have enough time to consider the value of visual inputs, which are silent and continuously available, and then process them selectively. As a result, the effect of auditory distractors on neural activity is shown in the early components, whereas the visual irrelevant information is collected in the late processing stage.

However, the three explanations above do not seem to be directly applicable to the influence of irrelevant information in auditory tasks, since the impacts and the moderation of modality are observed in behavioral performance and modeling parameters rather than neural activity (Stein, 2012). Nevertheless, the dynamic mechanism of the auditory categorical representation in the context of the intermodal or intramodal distractor is consistent with that of visual processing: the modality of irrelevant information modulates the impacts and both sensory processing and post-sensory decision processes are affected.

Specifically, the HDDM was implemented, and psychologically meaningful parameters were proposed to map various cognitive processes. Visual distractors resulted in a higher evidence accumulation rate and response boundary, eliciting higher response accuracy and indicating the impacts on both the sensory processing and post-sensory decision stages (Ratcliff and McKoon, 2008; Voss et al., 2015). In contrast, auditory distractors led to a lower evidence accumulation rate without affecting the response boundary, resulting in lower reaction time and indicating the impacts on the sensory processing rather than the post-sensory decision stage (Philiastides and Ratcliff, 2013; Sewell et al., 2018).

Taking the results of two tasks together, these findings pointed to modality-specific mechanisms of task-irrelevant information processing. The sensory processing stage of the visual categorical decision-making was affected by auditory distractors, whereas the post-sensory decision stage was affected by visual distractors. Similarly, the sensory processing stage of the auditory categorical decision-making were affected by auditory distractors whereas both stages were affected by visual distractors. These findings demonstrated the impacts of auditory distractors on the sensory processing stage while the impacts of the visual distractors on the post-sensory decision stage of visual categorical decision-making and both stages of auditory categorical decision-making.

The present study makes several noteworthy contributions. Theoretically and methodologically, the combination of modeling and neural data provided a comprehensive insight into which both the cognitive and neural mechanisms were disclosed (Frank et al., 2015; Turner et al., 2017; Delis et al., 2018).

This study appears to be a variant of the flanker task (Eriksen and Eriksen, 1974; Lavie, 1995), but in the current exploration, we primarily emphasize categorical decision-making. This research has two key differences from the flanker task. Firstly, the target to be categorized consisted of a deterministic feature and several probabilistic features, and the task-irrelevant information was congruent or incongruent with the deterministic feature of the target. The probabilistic features were less-relevant rather than irrelevant. Secondly, in the current design, the locations of the target features and the location of the task-irrelevant information were changing, and participants needed to search for the deterministic feature among the target’s various features for categorization. In the traditional flanker task, the target is defined by its spatial location, and three distracting stimuli are presented on either side of the target, flanking the central target stimulus (e.g., BBBABBB) (Merz et al., 2021). Overall, in line with the main purpose of the current research, this study focuses on categorical decision-making and aims to explore the impact of irrelevant information on visual and auditory categorical decision-making.

Several limitations to this study need to be acknowledged. First, while our findings are in line with prior categorization research (Matusz et al., 2015; Robinson et al., 2018), in which the behavioral performance of the congruent condition was not significantly different from the incongruent condition, and a previous study using the same tasks did not observe a congruency effect in behavioral performance either (Li and Deng, 2023b), there is insufficient evidence to suggest that the congruent and incongruent irrelevant inputs have the same impacts on the neural level or cognitive level. But we did not investigate its potential impacts due to its low relevance to the research questions, and a larger number of trials is preferable for evidencing the impacts of congruent or incongruent intermodal or intramodal task-irrelevant information in such categorical visual and auditory decision-making. Additionally, a direct comparison of the impacts of distractors on evidence accumulation between visual and auditory categorization was not performed, as the presentation method of the categorization stimuli was different between the two tasks (i.e., spatial separation in visual task vs. temporal separation in auditory task). Instead, the main purpose of the current study was to examine the modulation of distractor modalities on each task separately. Future research is needed to investigate the interaction between the target modalities and distractor modalities. Finally, since we did not identify any neural components of distractor impacts in the auditory task, it was not technically feasible to build the neural-directed modeling for auditory categorical decision-making. And we decided to interpret the results from different levels: the behavioral, cognitive (HDDM framework), and neural level. However, it could be possible that the impacts of irrelevant information on the evidence accumulation of auditory categorical decision-making were hard to detect at the neural level and innovations in techniques would be needed.

## 5 Conclusion

Taken together, this work provides a comprehensive understanding of multisensory categorical decision-making from an evidence accumulation perspective. We revealed the impacts of irrelevant inputs at the behavioral, cognitive, and neural levels, and demonstrated that irrelevant inputs influenced both the sensory processing and post-sensory decision stages of visual and auditory categorical decision-making, with modality-specific impacts on both the visual and auditory task. This study suggests the importance of considering modality-specific impacts when studying multisensory decision-making and contributes to our understanding of how humans process information from multiple sensory modalities.

## Data availability statement

Deidentified data and analysis scripts are available on the OSF repository (https://osf.io/vfegr/). Further inquiries can be directed to the first author Jianhua Li (christyjhli@um.edu.mo) and the corresponding author Sophia Deng (wdeng@um.edu.mo).

## Ethics statement

The studies involving humans were approved by Research Ethics Panel of the University of Macau. The studies were conducted in accordance with the local legislation and institutional requirements. The participants provided their written informed consent to participate in this study.

## Author contributions

JL: Conceptualization, Data curation, Formal analysis, Investigation, Methodology, Project administration, Validation, Visualization, Writing – original draft, Writing – review & editing. LH: Formal analysis, Methodology, Writing – review & editing. SD: Conceptualization, Formal analysis, Funding acquisition, Investigation, Methodology, Project administration, Resources, Supervision, Validation, Writing – original draft, Writing – review & editing.

## Footnotes

^1^This is a random factor to control the potential impacts of (1) the position of deterministic feature among the six features, (2) the variability of probabilistic features, whether they are the same or different, and (3) the congruency between task-relevant information and distractors. Thus, this factor has 24 levels (6 position × 2 variability × 2 congruency).

^2^http://www.python.org
